# Phenotypic and Molecular Characterization of ESBL-Producing Enterobacterales from Intestinal Colonization in Loja, Ecuador

**DOI:** 10.3390/antibiotics15100998

**Published:** 2026-10-09

**Authors:** Rosa Janneth Simaluiza-Masabanda, Blanca Pérez-Viso, Alisson Cambisaca, José Luis Chocho, José Miguel Romero-Saritama, Sofía Ochoa, Carmen Ullauri, Alexander Lizalde, Jeniffer Uchuari, Christian Jumbo, Heriberto Fernández, Rafael Cantón

**Affiliations:** 1 Department of Health Sciences, Universidad Técnica Particular de Loja, Loja 110101, Ecuador; sgochoa@utpl.edu.ec; 2 Servicio de Microbiología, Hospital Universitario Ramón y Cajal and Instituto Ramón y Cajal de Investigación Sanitaria (IRYCIS), 28034 Madrid, Spain; rafael.canton@salud.madrid.org; 3 CIBER de Enfermedades Infecciosas (CIBERINFEC), Instituto de Salud Carlos III, 28029 Madrid, Spain; 4 Degree Program in Biochemistry and Pharmacy, Faculty of Exact and Natural Sciences, Universidad Técnica Particular de Loja, Loja 110101, Ecuador; amcambisaca@utpl.edu.ec (A.C.); josettsu@gmail.com (J.L.C.); manuellizalde1997@gmail.com (A.L.); jenifferuchuari@gmail.com (J.U.); cajumbo3@utpl.edu.ec (C.J.); 5 Department of Biological and Exact Sciences, Universidad Técnica Particular de Loja, Loja 110101, Ecuador; jmromero@utpl.edu.ec; 6 Microbiology Laboratory, Isidro Ayora General Hospital, Loja 110103, Ecuador; carmen80_2000@yahoo.es; 7 Faculty of Human Health, National University of Loja, Loja 110101, Ecuador; 8 Institute of Clinical Microbiology, School of Medicine, Universidad Austral de Chile, Valdivia 5090000, Chile; hfernand@uach.cl

**Keywords:** extended-spectrum β-lactamases, Enterobacterales, *bla*_CTX-M_, coresistance, molecular surveillance, Ecuador

## Abstract

**Background/Objectives:** Extended-spectrum β-lactamase (ESBL)-producing Enterobacterales pose a significant threat to public health due to increasing antimicrobial resistance. In Ecuador, molecular characterization of these microorganisms remains limited. The objective of this study was to phenotypically and molecularly characterize ESBL-producing Enterobacterales in patients treated at a referral hospital in Loja, Ecuador. **Methods:** A descriptive cross-sectional study was conducted between November 2022–October 2023. 862 intestinal samples (stool samples and rectal swabs) were analyzed: 799 obtained from outpatient clinics, 34 from medical/surgical hospitalization wards, and 29 from the intensive care unit (ICU). Bacterial identification was performed using MALDI-TOF MS. Detection of ESBL and carbapenemase genes was performed using multiplex PCR. Phenotypic clustering was assessed by Fourier transform infrared spectroscopy (FT-IR). **Results:** A total of 195 (22.6%) ESBL-producing Enterobacterales were identified, of which 89 were molecularly characterized. *Escherichia coli* was the predominant species (79.8%), followed by *Klebsiella pneumoniae* (15.7%). High resistance rates to ciprofloxacin (57.1–100%) were observed being lower resistance to aminoglycosides (amikacin 1.5–57.1%; gentamicin 10.3–100%). The gene *bla*_CTX-M_ was the most frequent, detected in 79.8% of isolates, mainly in combination with *bla*_TEM-1_ (59.6%). Among the CTX-M family, the *bla*_CTX-M-1_ group was predominant (87.3%). Two isolates carried *bla*_KPC_. FT-IR analysis revealed species-dependent phenotypic clusters, particularly in *K. pneumoniae*, whereas *E. coli* showed greater spectral heterogeneity. **Conclusions:** ESBL-producing Enterobacterales were frequently identified among analyzed samples and exhibited a marked pattern of multidrug resistance, with a predominance of *bla*_CTX-M_ genes. These findings support the strengthening of molecular surveillance that might impact antimicrobial use.

## 1. Introduction

Extended-spectrum β-lactamases (ESBLs) represent one of the major challenges in clinical microbiology, due to their central role in antimicrobial resistance (AMR) to commonly used β-lactam antibiotics [1,2,3]. Produced predominantly by bacteria of the order Enterobacterales, these enzymes confer resistance to penicillins and third-generation cephalosporins, significantly limiting the available therapeutic options in both hospital and community settings [4,5].

The global dissemination of ESBL-producing Enterobacterales has become a major public health concern within the *One Health* framework, involving human, animal and environmental reservoirs, with reported prevalences ranging from 20–50% depending on the region and population studied [6,7]. In Latin America, reported rates of ESBL-producing *Escherichia coli* and *Klebsiella pneumoniae* exceed 30%, placing the region among the most affected worldwide [7]. This situation is associated with increased morbidity and mortality, higher healthcare costs, and greater complexity in clinical management [8].

In Latin America and the Caribbean, *E. coli* and *K. pneumoniae* are the predominant ESBL-producing Enterobacterales associated with both healthcare and community-acquired infections. In Ecuador, information on the prevalence and molecular epidemiology of ESBL-producing Enterobacterales remains limited. Previous studies conducted in Quito reported 22 ESBL-producing isolates among 143 clinical samples, with a predominance of *E. coli* (86.4%) and *Klebsiella oxytoca* (13.4%) [9]. Similarly, in the city of Loja, an ESBL prevalence of 81.6% was reported, with *E. coli* accounting for 82.2% of isolates, followed by *K. pneumoniae* (14.4%), *Proteus mirabilis* (2.2%), and *K. oxytoca* (1.1%) [10]. Together, these findings suggest a high local prevalence of ESBL-producing Enterobacterales and underscore the need for updated epidemiological studies integrating both phenotypic and molecular characterization.

According to the Ambler classification, ESBLs belong mainly to classes A and D, with the TEM, SHV, and CTX-M families representing the most clinically relevant groups [11]. Among these, CTX-M-type enzymes have become the most widespread family worldwide, particularly in *E. coli* isolates, and to a lesser extent in *K. pneumoniae* and other Enterobacterales, contributing to the emergence of community-acquired infections [2]. Their widespread dissemination, frequently mediated by mobile genetic elements, also facilitates the acquisition and co-occurrence of additional antimicrobial resistance determinants [2,3].

In recent years, Fourier transform infrared spectroscopy (FT-IR) has emerged as a rapid, reproducible, and low-cost phenotypic tool for phenotypic discrimination and epidemiological clustering of bacterial strains. The technique generates a spectral fingerprint derived mainly from cell wall polysaccharides and, in encapsulated species, from the composition of the capsular polysaccharide, allowing strains to be discriminated according to their phenotypic similarity. Although FT-IR does not replace high-resolution molecular typing methods such as whole-genome sequencing (WGS), multilocus sequence typing (MLST), or pulsed-field gel electrophoresis (PFGE), it has increasingly been proposed as a complementary first-line screening tool for outbreak detection and routine epidemiological surveillance in clinical microbiology laboratories, particularly for species such as *K. pneumoniae*, in which capsular variability notably influences the spectral profile [12,13,14].

In this context, the aim of this study was to phenotypically and molecularly characterize ESBL-producing Enterobacterales isolated from patients attending the Isidro Ayora General Hospital (HGIA) in Loja, Ecuador, and to explore the potential of FT-IR as a tool for the phenotypic clustering of these isolates.

## 2. Results

A total of 862 intestinal samples were collected, of which 195 (22.6%) yielded ESBL-producing Enterobacterales. The proportion of ESBL-producing Enterobacterales by healthcare setting was as follows: 22.4% (179/799) in outpatient clinics, 17.6% (6/34) in hospitalization wards, and 34.5% (10/29) in the ICU. Based on strain viability and recovery after storage, 89 phenotypically confirmed isolates were further used for subsequent molecular characterization.

To assess potential selection bias, the 89 molecularly characterized isolates were compared with the 106 non-characterized isolates (Supplementary Table S2). No significant differences were observed by sex (*p* = 0.637; Cramér’s V = 0.035) or age group (*p* = 0.100; Cramér’s V = 0.181). However, healthcare-setting distribution differed between groups (*p* = 0.043; Cramér’s V = 0.170), with ICU isolates accounting for 11.2% of the characterized group compared with 2.8% of the non-characterized group. Preliminary species identification of the non-characterized isolates was predominantly *E. coli* (84.9%), which was descriptively similar to the characterized group (79.8%).

### 2.1. Microbiological and Molecular Characterization of ESBL-Producing Enterobacterales

The species distribution showed a marked predominance of *E. coli* (71/89; 79.8%), followed by *K. pneumoniae* (14/89; 15.7%). *E. hormaechei* (3/89; 3.4%) and *K. oxytoca* (1/89; 1.1%) were identified less frequently.

**Figure 1:**
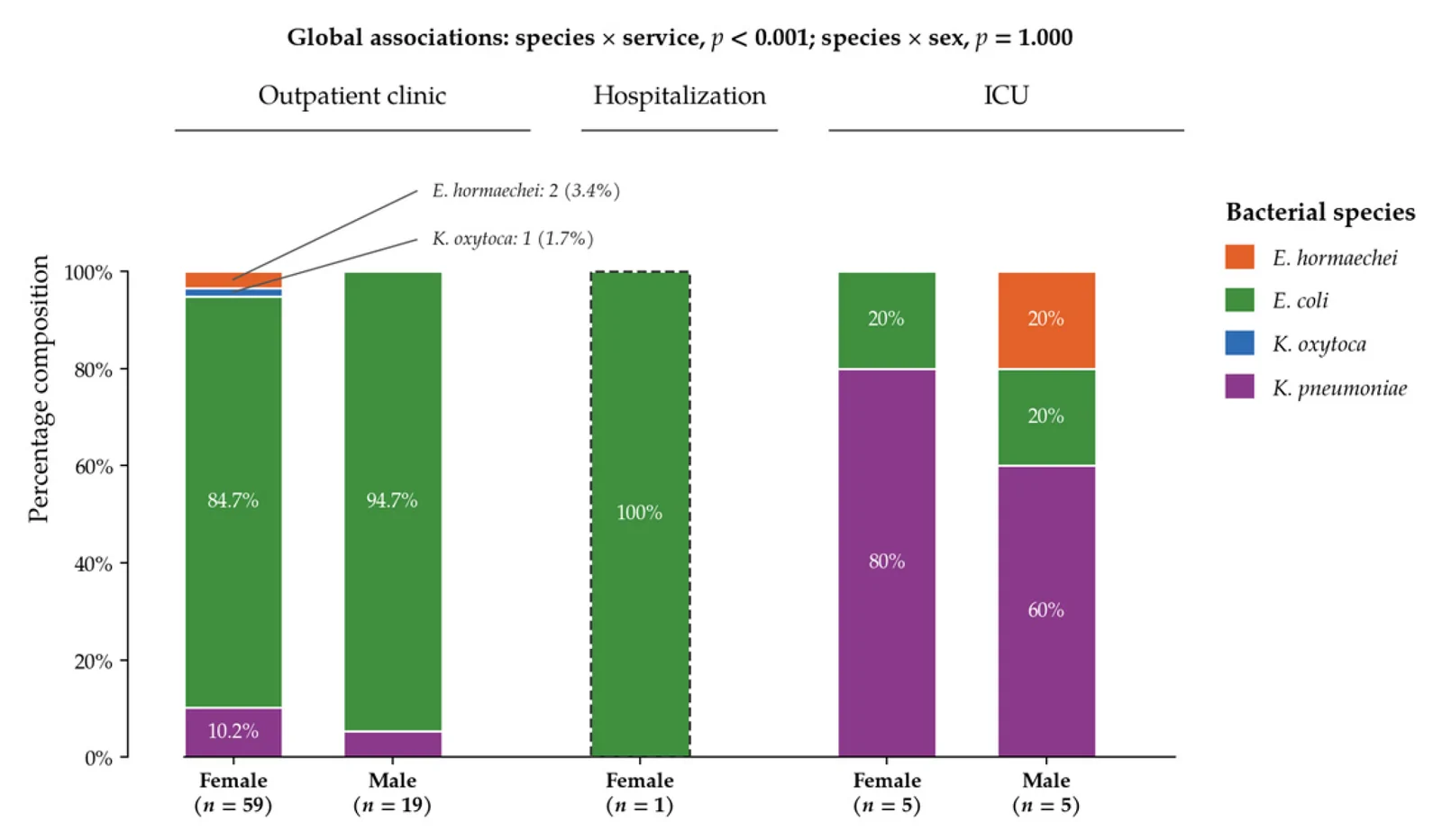
Distribution of ESBL-producing Enterobacterales species by healthcare setting and sex. Bars outlined with a dashed border represent strata with fewer than five isolates (Hospitalization–Female, *n* = 1); the percentage shown for this stratum is for descriptive purposes only.

As shown in Figure 1, bacterial species composition differed significantly by healthcare setting (*p* < 0.001; Cramér’s V = 0.395), but not by sex (*p* = 1.000; Cramér’s V = 0.071). *E. coli* predominated in the outpatient clinic irrespective of sex, whereas *K. pneumoniae* was more frequent among ICU patients, where *E. coli* and *E. hormaechei* were also identified. These findings suggest that differences in bacterial composition are mainly related to healthcare setting rather than to patient sex, although the small sample sizes in the hospitalization ward and ICU warrant cautious interpretation.

**Figure 2:**
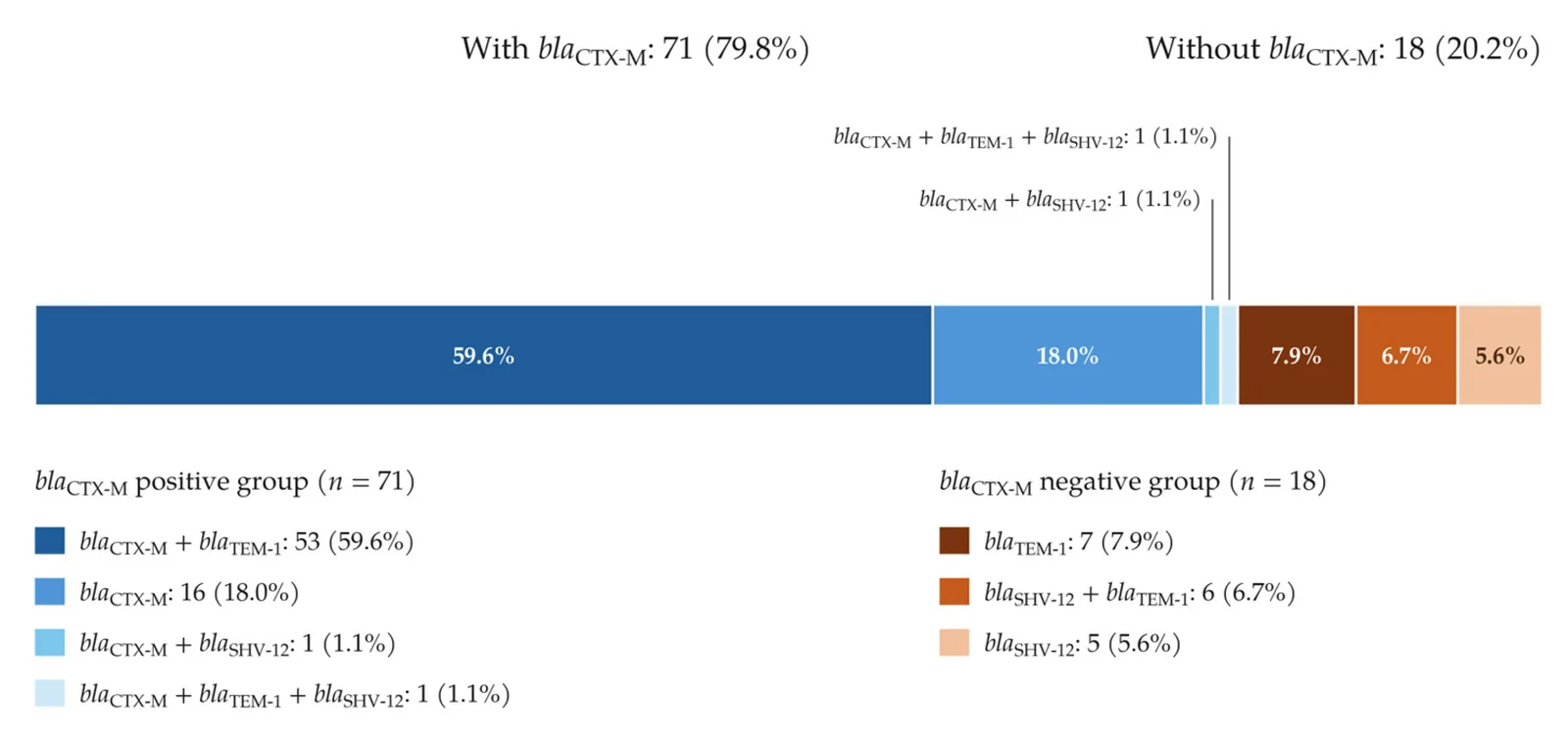
Distribution of ESBL genetic profiles in ESBL-producing Enterobacterales (*n* = 89).

Of the total ESBL-producing isolates (*n* = 89), *bla*_CTX-M_ was the most prevalent resistance determinant, identified in 71 samples (79.8%), with *bla*_CTX-M_ + *bla*_TEM-1_ being the most frequent combination (53/89; 59.6%), while its association with *bla*_SHV-12_ was less frequent (1/89; 1.1%). In contrast, *bla*_TEM-1_ and *bla*_SHV-12_ as single genes, without *bla*_CTX-M_, represented low proportions (7.9% and 5.6%, respectively) (Figure 2 and Table 1). It should be noted that *bla*_TEM-1_ is a narrow-spectrum β-lactamase and does not itself confer an ESBL phenotype; therefore, in isolates where it was the only β-lactamase gene detected, the molecular determinant underlying the observed ESBL phenotype remains unidentified. Two isolates additionally carried carbapenemase genes. One *K. pneumoniae* isolate harbored *bla*_SHV-12_ + *bla*_TEM-1_ and *bla*_KPC_, whereas one *E. hormaechei* isolate carried *bla*_CTX-M-1_ and *bla*_KPC_.

Among isolates carrying *bla*_CTX-M_, the CTX-M-1 group accounted for 87.3% of cases, while the CTX-M-9 group accounted for the remaining 12.7%.

No significant global association was observed between β-lactamase genetic profiles and sex (Fisher’s exact test, *p* = 0.649; Cramér’s V = 0.230). Global associations were observed between genetic profile and age group (*p* = 0.040; Cramér’s V = 0.298), healthcare setting (*p* = 0.014; Cramér’s V = 0.382), and bacterial species (*p* = 0.001; Cramér’s V = 0.349). Given the sparse distribution of several genetic profiles and the small numbers in some analytical strata, particularly hospitalization (*n* = 1), *E. hormaechei* (*n* = 3), and *K. oxytoca* (*n* = 1), these global associations should be interpreted as exploratory. The *bla*_CTX-M_ + *bla*_TEM-1_ profile predominated overall, particularly among *E. coli* isolates, whereas *K. pneumoniae* showed a greater representation of profiles containing *bla*_SHV-12_. *K. pneumoniae* isolates showed higher odds of carrying *bla*_SHV-12_ than *E. coli* isolates (8/14 [57.1%] vs. 5/71 [7.0%]; Fisher’s exact test, *p* < 0.001; OR = 16.54, 95% CI: 3.56–89.46) (Table 1).

### 2.2. Antimicrobial Resistance Profile of ESBL-Producing Enterobacterales by Bacterial Species and Healthcare Setting

As expected, the ESBL-producing Enterobacterales isolates exhibited a resistance pattern characterized by high rates against cefotaxime, ceftazidime, and aztreonam. In *E. coli* and *K. pneumoniae*, resistance to these antimicrobials exceeded 70%, particularly among ICU isolates. The lowest resistance rates (<70%) were observed for aminoglycosides (amikacin and gentamicin). Overall, resistance to ciprofloxacin was high (57.1–100%). Regarding healthcare setting, *K. pneumoniae* and *Enterobacter hormaechei* were not identified among hospitalized patients, while *Klebsiella oxytoca* was identified exclusively in the outpatient setting and was not recovered from hospitalized or ICU patients. The overall resistance profiles are detailed in Table 2.

### 2.3. Spectral Clustering Using Fourier Transform Infrared Spectroscopy (FT-IR) in ESBL-Producing Enterobacterales

**Figure 3:**
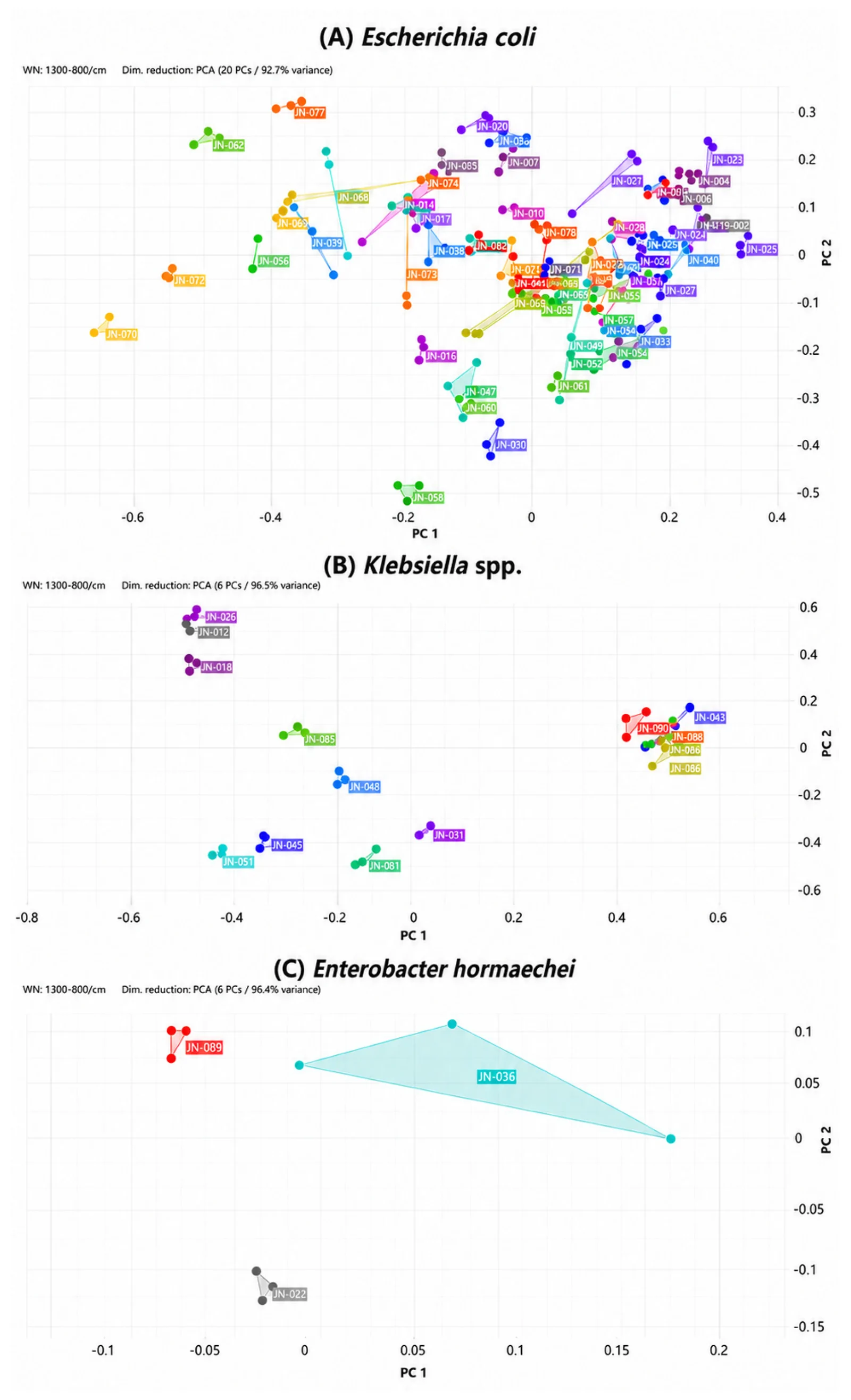
Species-stratified FT-IR/PCA of ESBL-producing Enterobacterales.

FT-IR-based spectral analysis was performed to explore phenotypic clustering among ESBL-producing Enterobacterales isolates. Spectra were analyzed using the 1300–800 cm^−1^ spectral region. Because the overall spectral distribution was influenced by species-level differences, subsequent interpretation was performed after stratification by species. Species-stratified PCA revealed distinct clustering patterns among the main taxa analyzed, with *E. coli*, *Klebsiella* spp., and *E. hormaechei* exhibiting distinct spectral distributions (Figure 3).

In contrast, *Klebsiella* spp. exhibited the most evident FT-IR clustering pattern, with several compact groups supported by consistent technical replicates and low intra-cluster distances. The most evident FT-IR-defined groups included clusters comprising the JN-012/JN-018/JN-026 and JN-083/JN-086/JN-087/JN-088 isolates. Additional isolates displayed reproducible but more isolated spectral profiles. Notably, isolate JN-090, the only carbapenemase (KPC) producer among the *Klebsiella* spp. isolates, did not form an independent spectral cluster but instead grouped closely with isolates JN-083, JN-086, JN-087, and JN-088, which produced ESBL enzymes exclusively. The distance matrix for *K. pneumoniae* isolates is presented in Figure S1 (Supplementary Materials).

Cluster assignment was based on the distance matrix and Bruker-defined FT-IR cluster allocation, whereas PCA was used as a visual representation of the spectral distribution. *E. coli* isolates exhibited a heterogeneous FT-IR profile, with multiple small FT-IR-defined groups and several reproducible individual profiles, but without a dominant spectral cluster. Isolates showing poor replicate consistency were not considered robust members of FT-IR-defined clusters.

Among *E. hormaechei* isolates, the analysis was considered descriptive due to the limited number of isolates. JN-022 and JN-089 showed reproducible but distinct FT-IR profiles, whereas JN-036 exhibited poor replicate consistency and was not assigned to a reliable FT-IR-defined cluster.

The main FT-IR-defined groups, their robust isolate members, and their associated ESBL gene profiles and origins are summarized in Table 3. Overall, FT-IR analysis revealed species-dependent spectral structure and several FT-IR-defined phenotypic clusters, particularly among *Klebsiella* spp. These clusters were compared with isolate origin and ESBL gene profiles as part of an exploratory analysis. In the absence of WGS, MLST or PFGE confirmation, FT-IR-defined groups were interpreted as phenotypic spectral clusters rather than confirmed clonal lineages.

## 3. Discussion

The present study provides an updated characterization of ESBL-producing Enterobacterales circulating at a tertiary-care hospital and the corresponding area of influence in southern Ecuador, combining phenotypic, molecular and FT-IR-based analyses to provide further insight into their epidemiology and resistance profiles.

With respect to intestinal carriage, the prevalence of intestinal carriage of ESBL-producing Enterobacterales was 22.6% (195/862), predominantly involving *bla*_CTX-M_-type genes. By healthcare setting, prevalence was 22.4% (179/799) among outpatients, 17.6% (6/34) among hospitalized patients, and 34.5% (10/29) among ICU patients. The outpatient prevalence found in this study is consistent with global estimates of community intestinal carriage of ESBL-producing *E. coli*, reported at 17.6% by Bezabih et al. [15] and 23.4% by Ng et al. [16]. Both studies also reported higher pooled prevalence in healthcare settings (21.1% and 27.7%, respectively), in line with the elevated prevalence observed among our ICU patients. These findings support intestinal colonization as a key reservoir for the dissemination of ESBL-producing Enterobacterales across both community and healthcare settings.

Regarding species distribution, *E. coli* was the predominant species among ESBL-producing isolates, followed by *K. pneumoniae*. This distribution is consistent with previous reports from the same institution [10] and with studies conducted in Ecuador and other Latin American countries [17,18,19]. The high prevalence of *E. coli* is consistent with the recognized ability of this species to acquire and disseminate antimicrobial resistance determinants, including those carried on mobile genetic elements. The widespread circulation of these resistance determinants among humans, animals, and environmental reservoirs underscores the importance of adopting a *One Health* approach to control the dissemination of ESBL-producing Enterobacterales [20].

When species were stratified by healthcare setting, *E. coli* predominated among outpatients, while *K. pneumoniae* was relatively more frequent in ICU patients. This distribution is consistent with the epidemiology of ESBL-producing Enterobacterales, in which *E. coli* stands out as a major cause of community-acquired infections, whereas *K. pneumoniae* is predominantly associated with healthcare-associated infections (HAIs), particularly in critically ill patients [6,20,21,22]. However, given the cross-sectional design of the study, these findings should be interpreted as differences in distribution by healthcare setting rather than as evidence of community or healthcare acquisition.

The antimicrobial susceptibility profile revealed high resistance to ciprofloxacin, particularly among ICU isolates, consistent with reports from Ecuador by Zurita et al. and Tusa-Torres et al. [17,18]. Fluoroquinolone resistance was not molecularly characterized in this study. This resistance phenotype typically arises from chromosomal mutations in *gyrA*/*parC*, often together with plasmid-mediated quinolone resistance (PMQR) determinants (*qnr*, *aac(*6*′)-Ib-cr*, *oqxAB*), some of which may be carried on plasmids also harboring *bla*_CTX-M_ and may contribute to their co-selection [23,24]. This potential co-selection mechanism could partly explain the elevated ciprofloxacin resistance observed in the present study. This finding is consistent with the frequent co-occurrence of ESBL production and fluoroquinolone resistance described in Enterobacterales, which may result from the co-selection and dissemination of multiple resistance determinants on mobile genetic elements [25,26]. Likewise, lower resistance was observed to aminoglycosides (amikacin and gentamicin), similar to that reported by Ochoa et al. [27], who reported that these antimicrobials retain greater activity against ESBL-producing Enterobacterales. This differential pattern is relevant for guiding empirical treatment selection, considering the acquired resistance criteria proposed by Magiorakos et al. [28].

Molecular characterization showed that *bla*_CTX-M_ was the predominant resistance determinant, consistent with previous studies conducted in Loja and Quito [9,10]. This finding reflects the global predominance of CTX-M-type ESBLs over TEM and SHV families, which have been extensively reported across human, healthcare, animal and food-associated reservoirs [19]. The *bla*_CTX-M_ + *bla*_TEM-1_ combination, previously reported in the local setting [10], suggests the circulation of mobile genetic elements that may facilitate the accumulation and dissemination of multiple resistance determinants [29,30]. Likewise, the detection of two isolates simultaneously carrying ESBL and *bla*_KPC_ genes highlights the coexistence of resistance mechanisms against broad-spectrum cephalosporins and carbapenems, which may substantially limit β-lactam treatment options.

Among isolates carrying *bla*_CTX-M_, the *bla*_CTX-M-1_ group predominated over the *bla*_CTX-M-9_ group, similar to what has been described in Latin America and Africa [31,32]. In contrast, greater diversity within the CTX-M family has been described in Europe and Asia [20,33,34], which may reflect differences in antimicrobial exposure, population structure, and the local dissemination of resistance-associated mobile genetic elements. Meanwhile, the higher frequency of *bla*_SHV_ in *K. pneumoniae* is consistent with reports from Ecuador and other countries, where this gene remains one of the main determinants of resistance in this species [19,20,32]. This finding further supports the recognized role of *K. pneumoniae* as an important nosocomial pathogen involved in the dissemination of multidrug resistance, particularly within intensive care units [19].

FT-IR analysis identified species-dependent phenotypic clustering patterns, with distinct spectral patterns in the 1300–800 cm^−1^ spectral region, associated with cell wall polysaccharides and bacterial capsule composition [12,13]. *E. coli* exhibited marked spectral heterogeneity, whereas *Klebsiella* spp. showed compact and reproducible clusters, likely reflecting, at least in part, differences in surface and capsular composition [13,14]. Although these groupings do not allow confirmation of clonal relationships or prediction of resistance gene profiles, the observed reproducibility of spectral clusters supports the potential use of FT-IR as a complementary screening approach for epidemiological surveillance [12,14], especially in *K. pneumoniae*. However, their utility for outbreak detection should be validated against high-resolution molecular typing in prospective studies. The results for *E. hormaechei* were purely descriptive due to the small number of isolates. These results support the potential of FT-IR as a rapid, reproducible, and cost-effective tool for the epidemiological surveillance of ESBL-producing Enterobacterales, complementary to higher-resolution techniques such as WGS, MLST, or PFGE.

Several limitations should be acknowledged. First, only the most common ESBL genes were characterized; therefore, other resistance mechanisms such as *bla*_OXA-1_ or porin deficiencies may have remained undetected. Second, the study was conducted at a single tertiary-care hospital, which may limit the generalizability of the findings. Third, only 89 of the 195 ESBL-producing isolates (45.6%) were available for molecular characterization. Nevertheless, the characterized and non-characterized groups were comparable with respect to sex and age distribution, although they differed by healthcare setting, with ICU isolates proportionally more frequent among characterized isolates. Fourth, the absence of high-resolution molecular typing techniques such as WGS or MLST prevented confirmation of the clonal relationships suggested by FT-IR clustering.

Finally, this study demonstrates the circulation of multidrug-resistant ESBL-producing Enterobacterales in Ecuador. These results emphasize the need to strengthen microbiological and molecular surveillance programs [35,36] and support the incorporation of rapid phenotypic tools such as FT-IR into epidemiological surveillance strategies. Such approaches, together with antimicrobial stewardship programs, infection prevention and control measures, may contribute to mitigating the spread of these high-risk pathogens [37,38].

## 4. Materials and Methods

### 4.1. Study Design and Ethical Considerations

A descriptive cross-sectional study was conducted at the Isidro Ayora General Hospital (HGIA) in the city of Loja, Ecuador, between November 2022 and October 2023. A total of 862 intestinal samples were analyzed from patients seen in outpatient clinics, during hospitalization, and admitted to the ICU.

This study was approved by the Bioethics Committee for Health Research at the University of Cuenca (code 2022-007EO-IE; 13 June 2022). Only adult patients who provided written informed consent were included. The information was treated confidentially and anonymously in accordance with the ethical principles of the Declaration of Helsinki.

### 4.2. Microbiological Processing and Bacterial Identification

Initial microbiological processing was performed at the Research Microbiology Laboratory of the Faculty of Health Sciences at the Universidad Técnica Particular de Loja (UTPL), Ecuador. The samples were inoculated onto CHROMagar™ ESBL and CHROMagar™ mSuperCARBA™ selective media (CHROMagar, Paris, France).

Rectal swabs were inoculated directly, while fecal samples were first diluted (1:10) in sterile saline. The plates were incubated at 37 °C ± 2 °C for 18–24 h under aerobic conditions.

Presumptive Enterobacterales colonies were re-inoculated onto blood agar (Becton, Dickinson and Company, Franklin Lakes, NJ, USA) and identified using the Enterosystem 18R system (Liofilchem, Roseto degli Abruzzi, Italy), following the manufacturer’s instructions. The identification of the isolates was confirmed by MALDI-TOF mass spectrometry (Bruker Daltonics, Bremen, Germany) at the Microbiology Service of Ramón y Cajal University Hospital (Madrid, Spain).

### 4.3. Inclusion and Exclusion Criteria

We included Enterobacterales isolates obtained from fecal or rectal swab samples collected from adult patients seen in outpatient clinics, inpatient units, and the ICU during the study period and with phenotypically confirmed ESBL production. Duplicate isolates from the same patient or non-Enterobacterales isolates were excluded. Only those that were viable after storage were further molecularly characterized.

Of the 195 ESBL-producing isolates initially recovered, 89 remained viable after storage and were available for molecular characterization, whereas 106 could not be recovered for confirmatory testing. To assess potential selection bias, characterized and non-characterized isolates were compared according to sex, age group, and healthcare setting. Species distributions were also summarized descriptively; however, formal comparison by species was not performed because different identification methods were used between groups (MALDI-TOF MS for characterized isolates and Enterosystem 18R for non-characterized isolates).

### 4.4. Antimicrobial Susceptibility Profile, Phenotypic Detection of ESBL, and Carbapenemases

Antimicrobial susceptibility testing was performed using the disk diffusion method on Difco™ Mueller-Hinton agar (Becton, Dickinson and Company). Ten antimicrobials were evaluated: ampicillin (AM, 10 µg), amoxicillin/clavulanic acid (AMC, 20/10 µg), piperacillin/tazobactam (TZP, 100/10 µg), cefotaxime (CTX, 30 µg), ceftazidime (CAZ, 30 µg), cefepime (FEP, 30 µg), aztreonam (ATM, 30 µg), amikacin (AK, 30 µg), gentamicin (CN, 10 µg), and ciprofloxacin (CIP, 5 µg). Antimicrobial discs were obtained from Bioanalyse (Ankara, Turkey), except for amoxicillin/clavulanic acid and aztreonam discs, which were obtained from Becton, Dickinson and Company. Results were interpreted as susceptible (S), intermediate (I), or resistant (R), according to the breakpoints for *Enterobacterales* in CLSI M100, 34th edition (2024) [39]. The interpretive breakpoints (susceptible, intermediate/SDD, resistant) applied for each antimicrobial are detailed in Supplementary Table S1. Fosfomycin was additionally tested using 50-µg discs but was excluded from the panel of interpretable antimicrobials, as CLSI M100 breakpoints for fosfomycin are validated specifically for the 200-µg disc supplemented with glucose-6-phosphate, and only for urinary isolates of *E. coli*; results could therefore not be categorically interpreted and are not included among the ten antimicrobials analyzed.

Phenotypic detection of ESBL was performed using the double-disc synergy test [40] and confirmed using combination discs of ceftazidime (CAZ, 30 µg) and cefotaxime (CTX, 30 µg), alone and in combination with clavulanic acid (10 µg) (Becton, Dickinson and Company). The synergy test was considered positive when a distortion or expansion of the inhibition zone toward the amoxicillin/clavulanic acid disc (AMC, 20/10 µg) was observed, corresponding to the “*keyhole effect*” [41]. For the combination discs, an isolate was considered ESBL-positive when the inhibition-zone diameter with clavulanic acid was ≥5 mm larger than the corresponding zone diameter of the antibiotic alone [41]. Phenotypic detection of carbapenemases was performed using the modified Hodge test with meropenem (MEM, 10 µg), considered positive when an arrowhead- or cloverleaf-shaped structure was observed. To differentiate serine (class A) from metallo-β-lactamase (class B) carbapenemases, synergy tests with boronic acid (APB, 300 µg) and EDTA (750 µg), respectively, were performed using meropenem and imipenem; synergy between the inhibitor and the carbapenem was considered positive. Meropenem and imipenem discs were obtained from Becton, Dickinson and Company, and EDTA discs from Bioanalyse. These phenotypic methods were considered complementary assays for characterizing carbapenem resistance mechanisms.

The interpretive criteria and phenotypic detection procedures were based on CLSI M100, 32nd edition (2022) [41].

*E. coli* ATCC 25922 (American Type Culture Collection, Manassas, VA, USA) was used as a quality control strain in antimicrobial susceptibility testing and phenotypic detection assays.

### 4.5. Molecular Detection of Resistance Genes

A total of 89 Enterobacterales isolates producing ESBL were selected for molecular analysis and sent to the Microbiology Department at Ramón y Cajal University Hospital (Madrid, Spain). The international shipment was authorized by Ecuador’s National Agency for Health Regulation, Control, and Surveillance (ARCSA) (authorization: ARCSA-ARCSA-CGTC-DTRSNSOYA-2024-4159-O).

The detection of ESBL genes (*bla*_TEM_, *bla*_SHV_ and *bla*_CTX-M_) and carbapenemases (*bla*_KPC_, *bla*_VIM_, *bla*_NDM_ and *bla*_OXA-48_) was performed by multiplex PCR using the primers and conditions detailed in Table 4. All reactions were performed using AmpliTaq Gold^®^ DNA Polymerase with Buffer II and MgCl (Applied Biosystems, Carlsbad, CA, USA).

The purified PCR product was subsequently sequenced by Sanger sequencing in Macrogen Europe (Macrogen Inc., Amsterdam, The Netherlands) using Applied Biosystems 3730XL DNA analyzers. 

### 4.6. FT-IR Analysis and Spectral Clustering of ESBL-Producing Enterobacterales

FT-IR was performed on 89 ESBL-producing Enterobacterales isolates using the IR Biotyper system (Bruker Daltonics, Bremen, Germany), following the manufacturer’s recommendations. Three technical replicates were obtained for each isolate. Spectral data were quality-controlled and preprocessed using the IR Biotyper Client Software (version 4.1.1.59) according to the manufacturer’s standard workflow. The spectral region from 1300–800 cm^−1^ was used for analysis. Spectral normalization and preprocessing were performed using the parameters implemented in the IR Biotyper software. Spectral data were analysed using principal component analysis (PCA) and hierarchical clustering analysis (HCA). PCA was used for visualization of the spectral distribution, whereas HCA was used to assess spectral relatedness. Euclidean distance was used as the distance metric, and hierarchical clustering was performed using average linkage. Cluster assignment was based on the distance matrix and the clustering algorithm implemented in the IR Biotyper software. Where applicable, the automatically calculated cut-off provided by the software was used to define spectral clusters. Analyses were first performed globally and subsequently stratified by species.

The resulting FT-IR-defined spectral clusters were compared with epidemiological and microbiological metadata, including isolate source and ESBL gene profile. Clusters were interpreted as phenotypic spectral groups suggestive of possible relatedness, without inferring definitive clonal relationships in the absence of confirmation by genomic or molecular typing.

### 4.7. Statistical Analysis

Statistical analyses were performed using R version 4.3.1. (R Foundation for Statistical Computing, Vienna, Austria). Categorical variables, including bacterial species distribution, sex, healthcare setting, age group, antimicrobial resistance profiles, and β-lactamase genetic profiles, were summarized as absolute frequencies and percentages, calculated using the corresponding number of isolates within each analytical stratum as the denominator.

Associations between categorical variables were evaluated using Fisher’s exact test, because several contingency tables contained small or zero cell counts. Global tests were performed to assess the association between bacterial species and healthcare setting, bacterial species and sex, and β-lactamase genetic profile and sex, age group, healthcare setting, or bacterial species. For R × C contingency tables with sparse observations, *p*-values were estimated using Monte Carlo simulation with 1,000,000 replicates. The strength of association in contingency tables was quantified using Cramér’s V statistic. For clinically relevant 2 × 2 comparisons, odds ratios (ORs) with corresponding 95% confidence intervals (95% CIs) were estimated using Fisher’s exact test. No post hoc pairwise comparisons were used to support the primary inferential conclusions; therefore, multiplicity adjustment was not applied to the global Fisher tests.

Because some analytical strata contained very few isolates, particularly the hospitalization stratum and less frequently represented bacterial species, estimates derived from small denominators were considered descriptive or exploratory and were interpreted cautiously. All statistical tests were two-sided, and statistical significance was defined as *p* < 0.05.

## 5. Conclusions

This study provides an updated characterization of ESBL-producing Enterobacterales at the Isidro Ayora General Hospital in Loja (Ecuador). *E. coli* was the predominant species, particularly among outpatients, whereas *K. pneumoniae* was more frequently recovered from ICU patients, supporting distinct epidemiological patterns of intestinal colonization between community and healthcare settings.

Isolates exhibited a marked MDR phenotype, with high resistance to β-lactam antibiotics, while aminoglycosides retained the highest in vitro activity. Molecular analysis confirmed the predominance of *bla*_CTX-M_, mainly in combination with *bla*_TEM-1_, highlighting the widespread circulation of these principal ESBL-associated determinants in this setting.

FT-IR analysis identified reproducible species-dependent spectral clusters, particularly among *K. pneumoniae*, supporting its potential as a rapid and cost-effective complementary tool for epidemiological surveillance. However, these spectral groupings should be interpreted as phenotypic clusters rather than confirmed clonal lineages in the absence of high-resolution molecular typing methods.

## Figures and Tables

**Table 1:** Distribution of genetic profiles according to clinical and epidemiological variables in ESBL-producing Enterobacterales (*n* = 89) *.

	β-Lactamase Gene Profile						
	*bla*_CTX-M_ ^†^	*bla_TEM-1_* ^‡^	*bla*_SHV-12_	*bla*_CTX-M_ ^†^ + *bla*_TEM-1_ ^‡^	*bla*_SHV-12_ + *bla*_TEM-1_ ^‡^	Other Combinations ^§^	Total
	*n* (%)	*n* (%)	*n* (%)	*n* (%)	*n* (%)	*n* (%)	*n* (%)
**Gender**							
Female	10 (11.2)	4 (4.5)	5 (5.6)	39 (43.8)	5 (5.6)	2 (2.2)	65 (73.0)
Male	6 (6.7)	3 (3.4)	0 (0.0)	14 (15.7)	1 (1.1)	0 (0.0)	24 (27.0)
**Total:**	**16 (18.0)**	**7 (7.9)**	**5 (5.6)**	**53 (59.6)**	**6 (6.7)**	**2 (2.2)**	**89 (100.0)**
**Age group**							
21–40 years	1 (1.1)	1 (1.1)	0 (0.0)	21 (23.6)	2 (2.2)	0 (0.0)	25 (28.1)
41–60 years	8 (9.0)	3 (3.4)	5 (5.6)	20 (22.5)	1 (1.1)	2 (2.2)	39 (43.8)
61–80 years	6 (6.7)	3 (3.4)	0 (0.0)	12 (13.5)	3 (3.4)	0 (0.0)	24 (27.0)
>80 years	1 (1.1)	0 (0.0)	0 (0.0)	0 (0.0)	0 (0.0)	0 (0.0)	1 (1.1)
**Total:**	**16 (18.0)**	**7 (7.9)**	**5 (5.6)**	**53 (59.6)**	**6 (6.7)**	**2 (2.2)**	**89 (100.0)**
**Healthcare Setting**							
CE	14 (15.7)	4 (4.5)	5 (5.6)	50 (56.2)	3 (3.4)	2 (2.2)	78 (87.6)
H	0 (0.0)	1 (1.1)	0 (0.0)	0 (0.0)	0 (0.0)	0 (0.0)	1 (1.1)
ICU	2 (2.2)	2 (2.2)	0 (0.0)	3 (3.4)	3 (3.4)	0 (0.0)	10 (11.2)
**Total:**	**16 (18.0)**	**7 (7.9)**	**5 (5.6)**	**53 (59.6)**	**6 (6.7)**	**2 (2.2)**	**89 (100.0)**
**ESBL-Producing Enterobacterales**							
*E. coli*	13 (14.6)	5 (5.6)	2 (2.2)	48 (53.9)	2 (2.2)	1 (1.1)	71 (79.8)
*K. pneumoniae*	2 (2.2)	2 (2.2)	3 (3.4)	2 (2.2)	4 (4.5)	1 (1.1)	14 (15.7)
*E. hormaechei*	1 (1.1)	0 (0.0)	0 (0.0)	2 (2.2)	0 (0.0)	0 (0.0)	3 (3.4)
*K. oxytoca*	0 (0.0)	0 (0.0)	0 (0.0)	1 (1.1)	0 (0.0)	0 (0.0)	1 (1.1)
**Total:**	**16 (18.0)**	**7 (7.9)**	**5 (5.6)**	**53 (59.6)**	**6 (6.7)**	**2 (2.2)**	**89 (100.0)**

*n*: number of strains; CE: outpatient clinic; H: hospitalization; ICU: intensive care unit. *: Values are presented as *n* (% of the total molecularly characterized isolates, *n*= 89). Percentages therefore do not represent within-row proportions. Categories with fewer than five isolates should be interpreted descriptively because of the limited denominator. ^†^: *bla*_CTX-M_ is reported at the group level (see Section 4.5 for sequencing detail). ^‡^: *bla*_TEM-1_ is a narrow-spectrum β-lactamase and does not by itself confer an ESBL phenotype; when detected as the sole gene (7.9% of isolates), the underlying ESBL determinant responsible remains unidentified. ^§^: “Other combinations” groups two rare gene-profile combinations, each detected in a single isolate (1.1%): *bla*_CTX-M_ + *bla*_SHV-12_, and *bla*_CTX-M_ + *bla*_TEM-1_ + *bla*_SHV-12_.

**Table 2:** Antimicrobial resistance profile of ESBL-producing Enterobacterales by bacterial species and healthcare setting (*n* = 89) * ^†^.

Identified	Healthcare		AM	AMC	TZP	CTX	CAZ	FEP	ATM	AK	CN	CIP
Bacteria	Setting	*n*	*n* (%)	*n* (%)	*n* (%)	*n* (%)	*n* (%)	*n* (%)	*n* (%)	*n* (%)	*n* (%)	*n* (%)
*Escherichia coli*	CE	68	60 (88.2)	29 (42.6)	6 (8.8)	67 (98.5)	60 (88.2)	45 (66.2)	53 (77.9)	1 (1.5)	7 (10.3)	58 (85.3)
	H	1	1 (100.0)	1 (100.0)	1 (100.0)	1 (100.0)	1 (100.0)	1 (100.0)	1 (100.0)	0 (0.0)	0 (0.0)	0 (0.0)
	ICU	2	2 (100.0)	1 (50.0)	0 (0.0)	2 (100.0)	2 (100.0)	2 (100.0)	2 (100.0)	0 (0.0)	0 (0.0)	2 (100.0)
*Klebsiella pneumoniae*	CE	7	7 (100.0)	1 (14.3)	1 (14.3)	7 (100.0)	6 (85.7)	1 (14.3)	5 (71.4)	1 (14.3)	4 (57.1)	4 (57.1)
	ICU	7	7 (100.0)	6 (85.7)	6 (85.7)	7 (100.0)	7 (100.0)	6 (85.7)	7 (100.0)	4 (57.1)	5 (71.4)	6 (85.7)
*Klebsiella oxytoca*	CE	1	1 (100.0)	1 (100.0)	0 (0.0)	1 (100.0)	1 (100.0)	0 (0.0)	1 (100.0)	0 (0.0)	0 (0.0)	1 (100.0)
*Enterobacter hormaechei*	CE	2	2 (100.0)	1 (50.0)	2 (100.0)	2 (100.0)	2 (100.0)	1 (50.0)	2 (100.0)	0 (0.0)	0 (0.0)	1 (50.0)
	ICU	1	1 (100.0)	1 (100.0)	0 (0.0)	1 (100.0)	1 (100.0)	0 (0.0)	1 (100.0)	0 (0.0)	1 (100.0)	0 (0.0)

*n*: number of strains; CE: outpatient clinic; H: hospitalization; ICU: intensive care unit. *: AM: ampicillin; AMC: amoxicillin/clavulanic acid; TZP: piperacillin/tazobactam; CTX: cefotaxime; CAZ: ceftazidime; FEP: cefepime; ATM: aztreonam; AK: amikacin; CN: gentamicin; CIP: ciprofloxacin. ^†^: Percentages were calculated using the number of isolates within each bacterial species × healthcare-setting stratum as the denominator. Percentages based on strata containing fewer than five isolates are presented for descriptive purposes only and should not be interpreted as precise resistance estimates. Strata meeting this criterion (*n* < 5) are shaded in gray throughout the table for easy identification.

**Table 3:** Summary of interpretable FT-IR-defined spectral groups and reproducibility assessment.

Species	FT-IR-Defined Group	Robust Isolate Profiles	Non-Robust/Excluded Isolates	Main ESBL Profile	Main Sample Origin	Interpretation
*E. coli*	Multiple small *E. coli* groups and individual profiles	Several small groups and reproducible individual profiles	Isolates with poor replicate consistency	Variable	Variable	High FT-IR heterogeneity; no dominant spectral cluster
*Klebsiella* spp.	Kpn-C1	JN-012/JN-018/JN-026	-	*bla*_SHV-12_	CE	Compact FT-IR-defined cluster
*Klebsiella* spp.	Kpn-C8	JN-083/JN-086/JN-087/JN-088	JN-043, if confirmed to be non-robust	*bla*_TEM-1_ and *bla*_SHV-12_ + *bla*_TEM-1_	ICU	Compact FT-IR-defined cluster
*E. hormaechei*	Reproducible profiles	JN-022/JN-089	JN-036	-	-	Descriptive; low number of isolates

**Table 4:** Primers and multiplex PCR conditions for the detection of ESBL and carbapenemase genes.

Genes	Primers	Sequence (5′–3′)	Amplicon (bp)	Conditions	Ref.
MULTIPLEX PCR TEM-SHV-CTX-M	SHV-F	ATGCGTTATATTCGCCTGTG	747	1 cycle of 5 min at 94 °C; 30 cycles of 1 min at 94 °C, 1 min at 60 °C, 2 min at 72 °C; final extension of 10 min at 72 °C.	[42]
	SHV-R	TGCTTTGTTATTCGGGCCAA			
	TEM-F	TCGCCGCATACACTATTCTCAGAATGA	445		
	TEM-R	ACGCTCACCGGCTCCAGATTTAT			
	CTX-M-F	ATGTGCAGYACCAGTAARGTKATGGC	593		
	CTX-M-R	TGGGTRAARTARGTSACCAGAAYCAGCGG			
MULTIPLEX PCR CTX-M-1 group/CTX-M-9 group	CTX-M-1G-F	AAAAATCACTGCGCCAGTTC	415	1 cycle of 5 min at 94 °C; 30 cycles of 25 s at 94 °C, 40 s at 52 °C, 50 s at 72 °C; final extension of 10 min at 72 °C.	[43]
	CTX-M-1G-R	AGCTTATTCATCGCCACGTT			
	CTX-M-9G-F	CAAAGAGAGTGCAACGGATG	205		
	CTX-M-9G-R	ATTGGAAAGCGTTCATCACC			
*bla*_CTX-M-1_ *	CTX-M-1-Seq-F	CCCATGGTTAAAAAATCACTGC	1000	1 cycle of 5 min at 94 °C; 30 cycles of 30 s at 94 °C, 30 s at 60 °C, 1 min at 72 °C; final extension of 10 min at 72 °C.	[44]
	CTX-M-1-Seq-R	CAGCGCTTTTGCCGTCTAAG			
*bla*_CTX-M-9_ *	CTX-M-9-Seq-F	TGGTGACAAAGAGAGTGCAACG	874	1 cycle of 5 min at 94 °C; 30 cycles of 30 s at 94 °C, 30 s at 55 °C, 40 s at 72 °C; final extension of 10 min at 72 °C.	[45]
	CTX-M-9-Seq-R	TCACAGCCCTTCGGCGAT			
MULTIPLEX PCR VIM-NDM-KPC- OXA-48	VIM-F	GATGGTGTTTGGTCGCATA	390	1 cycle of 5 min at 94 °C; 30 cycles of 30 s at 94 °C, 30 s at 52 °C, 1 min at 72 °C; final extension of 10 min at 72 °C	[46]
	VIM-R	CGAATGCGCAGCACCAG			
	OXA-48-F	GCGTGGTTAAGGATGAACAC	438		
	OXA-48-R	CATCAAGTTCAACCCAACCG			
	NDM-F	GGTTTGGCGATCTGGTTTTC	621		
	NDM-R	CGGAATGGCTCATCACGATC			
	KPC-F	CGTCTAGTTCTGCTGTCTTG	798		
	KPC-R	CTTGTCATCCTTGTTAGGCG			
*bla*_VIM_	VIM-seq-F	TATTGGTCTATTTGACCGCGT	800	1 cycle of 5 min at 94 °C; 30 cycles de 30 s at 94 °C, 30 s at 58 °C, 1 min at 72 °C; final extension of 10 min at 72 °C	[This study]
	VIM-seq-R	TACTCRRCGACTGAGCGAT			
*bla*_OXA-48_	OXA-48-seq-F	TTGGTGGCATCGATTATCGG	600	1 cycle of 5 min at 94 °C; 30 cycles of 30 s at 94 °C, 30 s at 56 °C, 1 min at 72 °C; final extension of 10 min at 72 °C	[47]
	OXA-48-seq-R	GAGCACTTCTTTTGTGATGGC			
*bla*_NDM_	NDM-seq-F	AAGCTGAGCACCGCATTA	713	1 cycle of 5 min at 94 °C; 30 cycles of 30 s at 94 °C, 30 s at 52 °C, 1 min at 72 °C; final extension of 10 min at 72 °C	[48]
	NDM-seq-R	GCTCATCACGATCATGCT			
*bla*_KPC_	KPC-seq-F	ATGTCACTGTATCGCCGTC	800	1 cycle of 5 min at 94 °C; 30 cycles of 30 s at 94 °C, 30 s at 54 °C, 1 min at 72 °C; final extension of 10 min at 72 °C	[49]
	KPC-seq-R	TTACTGCCCGTTGACGCC			

ESBL genes: *bla*_TEM_, *bla*_SHV_, *bla*_CTX-M_. Carbapenemase genes: *bla*_KPC_, *bla*_VIM_, *bla*_NDM_, *bla*_OXA-48_. *: Isolates positive for *bla*_CTX-M_ by group-specific multiplex PCR were confirmed by Sanger sequencing of the group-specific amplicon (CTX-M-1 group/CTX-M-9 group primers), which verified group assignment but did not resolve the specific allelic variant; therefore, *bla*_CTX-M_ is reported without variant-level designation. In contrast, *bla*_TEM_ and *bla*_SHV_ amplicons were fully sequenced, allowing variant-level identification (*bla*_TEM-1_ and *bla*_SHV-12_, respectively).

## Data Availability

The data supporting the results presented in this study are available upon reasonable request to the corresponding authors. The data are not publicly available due to ethical and confidentiality restrictions regarding the participants.

## Supplementary Materials

The following supporting information can be downloaded at https://www.mdpi.com/article/10.3390/antibiotics15100998/s1, Figure S1: Distance matrix of ESBL-producing *K. pneumoniae* isolates based on FT-IR spectra; Table S1: Interpretive breakpoints (inhibition zone diameter, in mm) used for the phenotypic interpretation of antimicrobial susceptibility in ESBL-producing Enterobacterales isolates [39]; Table S2: Comparison between molecularly characterized (*n* = 89) and non-characterized (*n* = 106) ESBL-positive isolates, to assess potential selection bias.

## Abbreviations

The following abbreviations are used in this manuscript:

AK	Amikacin
AM	Ampicillin
AMC	Amoxicillin/clavulanic acid
ARCSA	National Agency for Health Regulation, Control, and Surveillance
ATM	Aztreonam
CAZ	Ceftazidime
CE	Outpatient clinic
CIP	Ciprofloxacin
CLSI	Clinical and Laboratory Standards Institute
CN	Gentamicin
CTX	Cefotaxime
ESBL	Extended-spectrum β-lactamases
FEP	Cefepime
FT-IR	Fourier transform infrared spectroscopy
H	Hospitalization
HGIA	Isidro Ayora General Hospital
ICU	Intensive Care Unit
MDR	Multidrug-resistant microorganisms
MLST	Multilocus Sequence Typing
n	Number of isolates
PFGE	Pulsed-Field Gel Electrophoresis
TZP	Piperacillin/tazobactam
WGS	Whole-genome sequencing
