# Supplementary material for: Phenotypic and Molecular Characterization of ESBL-Producing Enterobacterales from Intestinal Colonization in Loja, Ecuador
Source: Antibiotics (Basel). 2026 Oct 9;15(10):998. doi: 10.3390/antibiotics15100998 (PMC13700656; doi:10.3390/antibiotics15100998)
Supplement: Supplementary file 1 [file antibiotics-15-00998-s001.zip › Table S1. Breakpoints_ESBL_Enterobacterales.pdf]

Table S1. Interpretive breakpoints (inhibition zone diameter, in mm) used for the phenotypic interpretation of antimicrobial susceptibility in ESBL-producing Enterobacterales isolates.

Antimicrobial	Susceptible (S)	Intermediate (I) / SDD	Resistant (R)
Ampicillin (AM)	≥17 mm	14–16 mm	≤13 mm
Amoxicillin/clavulanic acid (AMC)	≥18 mm	14–17 mm	≤13 mm
Piperacillin/tazobactam (TZP)	≥25 mm	SDD 21–24 mm	≤20 mm
Cefotaxime (CTX)	≥26 mm	23–25 mm	≤22 mm
Ceftazidime (CAZ)	≥21 mm	18–20 mm	≤17 mm
Cefepime (FEP)	≥25 mm	SDD 19–24 mm	≤18 mm
Aztreonam (ATM)	≥21 mm	18–20 mm	≤17 mm
Amikacin (AK)	≥20 mm	17–19 mm	≤16 mm
Gentamicin (CN)	≥18 mm	15–17 mm	≤14 mm
Ciprofloxacin (CIP)	≥26 mm	22–25 mm	≤21 mm

Abbreviations: S: susceptible; I: intermediate; SDD: susceptible-dose dependent; R: resistant.
Source: CLSI M100, 34th edition (2024) [39].
