# Supplementary material for: Phenotypic and Molecular Characterization of ESBL-Producing Enterobacterales from Intestinal Colonization in Loja, Ecuador
Source: Antibiotics (Basel). 2026 Oct 9;15(10):998. doi: 10.3390/antibiotics15100998 (PMC13700656; doi:10.3390/antibiotics15100998)
Supplement: Supplementary file 1 [file antibiotics-15-00998-s001.zip › Table S2. Comparison of ESBL-Positive Isolates by Molecular Characterization.pdf]

Table S2. Comparison between molecularly characterized (*n* = 89) and non-characterized (*n* = 106) ESBL-positive isolates, to assess potential selection bias.

Variable	Characterized (*n* = 89)	Non-characterized (*n* = 106)	*p*-value *	Cramér's V
**Sex**				
Female	65 (73.0)	74 (69.8)		
Male	24 (27.0)	32 (30.2)	0.637	0.035
**Healthcare setting**				
Outpatient clinic	78 (87.6)	101 (95.3)		
Hospitalization	1 (1.1)	2 (1.9)		
ICU	10 (11.2)	3 (2.8)	0.043	0.170
**Age group †**				
21–40 years	25 (28.1)	24 (23.5)		
41–60 years	39 (43.8)	39 (38.2)		
61–80 years	24 (27.0)	30 (29.4)		
>80 years	1 (1.1)	9 (8.8)	0.100	0.181
**Species identification ‡**				
E. coli	71 (79.8)	90 (84.9)	-	-
K. pneumoniae	14 (15.7)	2 (1.9)	-	-
K. oxytoca	1 (1.1)	1 (0.9)	-	-
E. hormaechei	3 (3.4)	0 (0.0)	-	-
Other/no data	0 (0.0)	13 (12.3)	-	-

* Fisher's exact test (sex) or Monte Carlo simulation with 1,000,000 replicates for sparse R×C tables (healthcare setting, age group), consistent with the statistical analysis described in Methods.

† Excludes 4 isolates under 21 years, all belonging to the non-characterized group; this category is therefore absent among characterized isolates.

‡ Identification by MALDI-TOF (characterized) vs. Enterosystem 18R (non-characterized, preliminary method); as these are not equivalent, the comparison is descriptive only.
